# Hybrid Nanoparticles for Haloperidol Encapsulation: Quid Est Optimum?

**DOI:** 10.3390/polym13234189

**Published:** 2021-11-30

**Authors:** Sergey K. Filippov, Ramil R. Khusnutdinov, Wali Inham, Chang Liu, Dmitry O. Nikitin, Irina I. Semina, Christopher J. Garvey, Shamil F. Nasibullin, Vitaliy V. Khutoryanskiy, Hongbo Zhang, Rouslan I. Moustafine

**Affiliations:** 1 Pharmaceutical Sciences Laboratory and Turku Bioscience Center, Åbo Akademi University, 20520 Turku, Finland; wali.inam@abo.fi (W.I.); chang.liu@abo.fi (C.L.); hongbo.zhang@abo.fi (H.Z.); 2 Turku Bioscience Center, University of Turku, 20520 Turku, Finland; 3 School of Pharmacy, University of Reading, Whiteknights, Reading RG6 6AD, UK; v.khutoryanskiy@reading.ac.uk; 4 Institute of Pharmacy, Kazan State Medical University, 16 Fatykh Amirkhan, 420126 Kazan, Russia; ramil7316@gmail.com (R.R.K.); shamil.nasibullin@gmail.com (S.F.N.); 5 Department of Pharmacology, Kazan State Medical University, 49 Butlerov str., 420012 Kazan, Russia; richard4777@yandex.ru (D.O.N.); seminai@mail.ru (I.I.S.); 6 Heinz Maier-Leibnitz Zentrum (MLZ), Technische Universität München, Lichtenbergstraße 1, 85748 Garching, Germany; christopher.garvey@tum.de

**Keywords:** Eudragits, haloperidol, antipsychotic drug, nanoparticles, hybrid, SANS, ITC, TEM, mDSC, DLS, MSN

## Abstract

The choice of drug delivery carrier is of paramount importance for the fate of a drug in a human body. In this study, we have prepared the hybrid nanoparticles composed of FDA-approved Eudragit L100-55 copolymer and polymeric surfactant Brij98 to load haloperidol—an antipsychotic hydrophobic drug used to treat schizophrenia and many other disorders. This platform shows good drug-loading efficiency and stability in comparison to the widely applied platforms of mesoporous silica (MSN) and a metal–organic framework (MOF). ZIF8, a biocompatible MOF, failed to encapsulate haloperidol, whereas MSN only showed limited encapsulation ability. Isothermal titration calorimetry showed that haloperidol has low binding with the surface of ZIF8 and MSN in comparison to Eudragit L100-55/Brij98, thus elucidating the striking difference in haloperidol loading. With further optimization, the haloperidol loading efficiency could reach up to 40% in the hybrid Eudragit L100-55/Brij98 nanoparticles with high stability over several months. Differential scanning calorimetry studies indicate that the encapsulated haloperidol stays in an amorphous state inside the Eudragit L100-55/Brij98 nanoparticles. Using a catalepsy and open field animal tests, we proved the prolongation of haloperidol release *in vivo*, resulting in later onset of action compared to the free drug.

## 1. Introduction

Hybrid nanoparticles such as mesoporous silica and metal–organic frameworks have attracted attention in the recent years due to possibility of high drug loading in comparison with classical drug delivery carriers such as polymeric micelles [1,2], liposomes [3,4], etc. Metal–organic frameworks (MOFs) made of metal clusters and organic moieties have a variety of benefits including precise 3D structure with significant porosity and the feasibility of synthetic routes to combine metallic and organic elements in one structure [5,6,7,8] Significant progress was achieved during the last decade to exploit MOFs as a drug delivery carrier making it a prospective platform in nanomedicine [9,10,11,12].

Inorganic carriers such as mesoporous silica nanoparticles (MSNs) have a porous surface which have gained MSNs the attention of drug delivery community. The chemical and physical properties of MSNs can be tuned and controlled with high precision. By modifying the surface of MSN it is possible to increase or decrease the surface area adjusting the drug dosage and release [13,14,15,16,17] MSNs have proven to be very biocompatible and non-toxic in humans [18].

Amphiphilic block and random copolymers have been studied and utilized in a plethora of applications as drug/protein/gene delivery nanocarriers for more than three decades due to their ability to self-assemble in a variety of nanostructures in aqueous milieu [19,20]. Their final characteristics are dictated by certain physicochemical parameters such as solubilization protocol, the nature of constituent monomers, molecular weight, etc. The size and shape of nanoparticles and drug loading can be tuned by variation of polymer composition. Stimuli-responsive polymers possess the ability to alter their chain conformation upon the changes of certain external stimuli, such as pH [20,21], temperature [22,23], light [24]. It is of paramount importance to use non-toxic materials when developing drug delivery carriers; the usage of the polymer that is approved by FDA or other similar agencies would be the best way to achieve potential therapeutic future. The essential difference between “approved” and “non-approved” polymers from the market point of view can be illustrated by the drastic difference between the number of papers reporting polymeric drug delivery systems and the number of polymer-based drugs available on a market nowadays [25,26].

Nano- and microcontainers based on FDA approved acrylic and methacrylic acid-based pH-sensitive polymers commercially known as Eudragits are widely used in drug delivery area [27,28,29,30]. Eudragits applications vary from encapsulation of some enzyme-based drugs [31,32] to nasal, [28,30] oral [29] and intestinal drug delivery [33,34].

Previously the formation of Eudragit L100-55 nanoparticles in the presence of the nonionic surfactant Brij98 was studied [35]. It was reported that addition of a certain amount of surfactant Brij98 to polymer Eudragit L100-55 provides the possibility to prepare stable nanoparticles and prevent a macroscopic polymer phase separation at acidic pH. A trypsin delivery system based on Eudragit/Brij98 nanoparticles successfully encapsulated trypsin in Eudragit/Brij98 nanocontainers preventing its deactivation at different pH values [35]. In this work we examine Eudragit/Brij98 nanocontainers as drug delivery carriers for the encapsulation of haloperidol. Haloperidol is an antipsychotic drug with very poor solubility in water of 14 µg/mL [36,37,38]. Several approaches to overcome haloperidol low solubility were suggested recently [36,39]. Enhanced solubility of haloperidol up to 0.3 mg/mL by adding glutaric acid was reported [39]. The presence of lactic acid also improves the solubility of haloperidol [36]. The traditional routes to encapsulate haloperidol such as lipid or polymer-based formulations have been extensively tested over the last decades [40,41,42].

In summary, MSN, MOF, and polymeric nanoparticles proved to be effective and biocompatible platforms for drug encapsulation given rise to the question: *quid est optimum*?—which platform works the best for haloperidol encapsulation? In order to provide an answer to this specific question, and a more general methodology to answer this question for different kinds of polymeric nanoparticles, we present a physical characterisation as a rationalisation for the behaviour of this formulation in an animal system. The quest for the best drug carrier is the main motivation of this paper having haloperidol as a model drug to have rigorous comparison of different platforms for encapsulation.

## 2. Materials

Eudragit L100-55 (methacrylic acid-*co*-ethyl acrylate) 1:1) was obtained from Evonik Röhm GmbH (Darmstadt, Germany) as a gift and was used as an acidic polymer. The molecular weight of Eudragit L100-55 is 250,000.0 g/mole. All other chemicals were purchased from Sigma-Aldrich Ltd. Haloperidol (purity > 98%) was purchased both from Sigma-Aldrich Ltd. and Gedeon-Richter, Budapest, Hungary. The purity of other compounds was above 99%.

### 2.1. Preparation of the ZIF-8 Nanoparticles

The ZIF-8 was synthesized by referring to the literature method [8] with minor modifications. Typically, 0.2 g Zn(NO_3_)_2_·6H_2_O was dissolved in methanol (MeOH, 0.8 mL). Then, the prepared Zn(NO_3_)_2_ solution was mixed with 10 mL of ferrocene solution in MeOH under vigorous stirring. After 5 min, 2-methylimidazole (MIM) (0.2 g) was added dropwise and stirred for a further 30 min to prepare the yellow ZIF-8 nanoparticles. The precipitates were washed three times.

### 2.2. Synthesis of Mesoporous Silica Nanoparticles

The synthesis of the MSN-NH_2_ was carried out by using a protocol previously reported in the literature [18]. Specifically, MSN-NH_2_ were prepared by the co-condensation procedure where APTES and TEOS served as the precursor of silica. To synthesize the MSN-NH_2_, a solution of cetyltrimethylammonium bromide (CTAB, 1.17 g, serving as a template for the silica condensation) in a solution of 180 mL of water and 30 mL of ethylene glycol is heated to 50 °C in a three-neck round bottom flask of 250 mL capacity. When this temperature was reached, ammonium hydroxide (30 wt%, 5.45 mL, serving as a catalyst for the silica condensation), tetraethoxysilane (TEOS, 2.81 mL) and the aminoethylethoxysilane (APTES, 0.327 mL) were mixed and added dropwise to the solution under stirring. The mixture continues under stirring for 2 h at 50 °C. Then, stirring was stopped and the colloidal solution was aged at 50 °C overnight. Afterwards, heating was stopped, and the solution was allowed to cool down. At the end, the MSN-NH_2_ were purified using centrifugation (10,000 g, 10 min) and resuspended in a “template free” solution, containing 20 g ammonium nitrate in 1L of ethanol. This centrifugation and resuspension process is performed three times and the final product were resuspended in methanol for further use.

### 2.3. Preparation of Eudragit/Brij98 Nanoparticles with Loaded Haloperidol

In this study we utilized the protocol for the assembly of Eudragit/Brij98 nanoparticles reported in our previous work modified to achieve high drug loading of haloperidol [35]. Encapsulation of haloperidol was performed as follows: 2.1 mg of Eudragit L100-55 and 0.7 mg of Brij98 were dissolved in 1 mL of purified water, the pH was adjusted to 11.0, and the solution was stirred for 1 h. Haloperidol solution in acetone with 1, 2, or 4 mg/mL was added to the solution and stirred for 10 min, and then the pH was adjusted to 3.0−5.0. The formation of nanoparticles can be easily monitored from the solution opalescence. Acetone was removed on rotary evaporator RV 10 control V auto (IKA^®^ Werke GmbH, Staufen, Germany) as the final step.

### 2.4. Physicochemical Characterisation

Dynamic Light Scattering. DLS measurements were recorded using a Zetasizer NanoZS (Malvern Instruments Ltd., Worcestershire, UK). All readings were repeated three times for each measurement at 100 s per run. The DTS (Nano) program was used to evaluate the data. Intensity-weighted hydrodynamic diameter *D*_h_ distribution functions were used for the analysis of intensity correlation function from the solutions.

Transmission Electron Microscopy. The surface morphology of unloaded EugragitL100-55/Brij98, EugragitL100-55/Brij98 loaded with haloperidol was evaluated by using the transmission electron microscope (TEM; JEOL 1400 Plus, JEOL, Peabody, MA, USA). The samples for TEM studies were prepared by dropping 20 µL of nanoparticle suspension to a grid placed on a piece of filter paper to remove the excess of the solution. The sample was dried overnight. No staining was used. The applied voltage was varying from 80 to 200 keV depending on the required resolution.

Thermal Analysis. Modulated DSC (mDSC) measurements were carried out using a Discovery DSC™ (TA Instruments, New Castle, DE, USA), equipped with a refrigerated cooling system (RCS90). TRIOS™ software (version 3.1.5.3696) was used to analyse the DSC data (TA Instruments, New Castle, DE, USA). Tzero aluminium pans (TA Instruments, New Castle, DE, USA) were used in all calorimetric studies. The empty pan was used as a reference and the mass of the reference pan and of the sample pans were taken into account. Dry nitrogen was used as a purge gas through the DSC cell at 50 mL/min. Indium and n-octadecane standards were used to calibrate the DSC temperature scale; enthalpic response was calibrated with indium. Calibration of heat capacity was done using sapphire. Initially the samples were cooled from room temperature to 0 °C, then kept at 0 °C for 5 min and analysed from 0 to 200 °C. The modulation parameters used were: 2 °C/min heating rate, 40 s period and 0.212 °C amplitude. Glass transition temperatures were determined using the reversing heat flow signals. All measurements were performed in triplicate.

Fourier Transformed Infrared (ATR-FTIR) Spectroscopy. ATR-FTIR spectra were recorded by a Nicolet iS5 FTIR spectrometer (Thermo Scientific, Waltham, MA, USA) using the iD5 smart single bounce ZnSe ATR crystal. The spectra were analysed using OMNIC spectra software.

Isothermal titration calorimetry (ITC). The microcalorimetry study was performed using a MicroCal iTC200 (Malvern Instruments Ltd., Worcestershire, UK) isothermal titration calorimeter. The experiment was performed with consecutive injections of the haloperidol solution (c = 1 mg/mL) into the 280 μL calorimeter cell with the solution containing hybrid material (ZIF8, MSN, or Eudragit L100-55). The haloperidol solution was added to a 40 μL injection syringe, the tip of which was modified to act as a stirrer. The stirring speed was 1000 rpm. The injection volume was 2 μL in each experiment. The time between injections was 200 s. The enthalpograms were analyzed by OriginLab Software or NITPIC [43,44].

SANS measurements. Sample solutions were loaded into cylindrical 2 mm quartz cuvettes (Hellma GmbH, Müllheim, Germany) for small angle neutron scattering (SANS) measurements. SANS measurements were carried out on the fixed wavelength pinhole instrument QUOKKA [45] at the Australian Centre for Neutron Scattering (ANSTO, Lucas Heights Australia). Structural information is extracted from the absolutely scaled intensity, *I*, versus scattering vector, *q*, data where *q* = 4π sin (θ)/λ where 2*θ* is the scattering angle and λ is the neutron wavelength. Raw 2D SANS patterns were collected at 2 sample to detector distances, 2 and 14 m, for 2000 and 6000 s, respectively. The raw 2D data were converted absolutely normalised scattering cross-section, *I*(*q*), using the QUOKKA specific macros based on the NIST data reduction macros [46] written for IgorPro (Wavemetric, Oswego, NY, USA). After first subtracting background signal due the cadmium blocked beam (dark current of neutrons and electronic noise), and then subtracting the scattering due to the empty quartz cuvette scaled by the sample and cuvette transmission, the measurement geometry is converted to radially averaged *I*(*q*). *I*(*q*) was scaled to the flux of the incident beam to provide the absolutely scaled scattering cross-section covering a range of scattering vectors 5.7 × 10^−3^ < *q* < 0.4 Å^−1^.

### 2.5. In Vivo Catalepsy Recording Test

#### 2.5.1. Animals

In vivo experiments were carried out on 40 male Wistar rats weighing 180–200 g. Before the start of the experiments, all animals were kept in standard vivarium conditions with a natural light regime on a complete balanced diet (GOST R 50258-92) in compliance with the International Recommendations of the European Convention for the Protection of Vertebrate Animals used in Experimental Research (1997), the Rules of Good Laboratory Practice, approved by the order of the Ministry of Health of the Russian Federation No. 199n dated 1 April 2016. All studies were approved by the Ethical Review Committee of Kazan State Medical University (Protocol No. 8 of 30 October 2018).

#### 2.5.2. Behavioural Techniques

To analyse the intranasal effect of haloperidol, behavioural techniques were used that reflect the pharmacological properties characteristic of antipsychotics (Miyamoto S., 2005).

*(a) The cataleptogenic effect of haloperidol.*

Cataleptogenic action (catalepsy), i.e., the ability of an animal to hold an artificially given posture, is one of the manifestations of undesirable side effects of extrapyramidal drugs with neuroleptic activity [47]. Nanoparticles with haloperidol were tested in 8 animals. A commercial formulation of haloperidol (5 mg mL of sterile solution containing lactic acid, Gedeon-Richter, Budapest, Hungary) was used in control experiments.

Nanoparticles with haloperidol or control (the dose of haloperidol from all samples was 1 mg/kg of rat weight) were instilled into the nostrils of each rat for 5 min using a specially designed plastic cannula, and then each animal was placed in a special setup for assessing catalepsy (Open Science, Moscow, Russia) with a plastic bar at a height of 10 cm as described in ref [42]. The animals were carefully placed on the bar at 10, 30, 60, 120, and 180 min after intranasal administration of nanoparticles with haloperidol or a control solution of haloperidol, and the ability to maintain a lecturer’s posture was recorded (the duration on the bar) for 180 s. If the duration of the stay of the rat on the bar reached 180 s, it was carefully removed. After each test, the animals were returned to their home cages.

*(b) Behaviour in the “Open Field”*.

The study of the effect of intranasal administration of haloperidol at a lower dose (0.5 mg/kg) as a part of the formulation was carried out using the «Open Field» method [48], which makes it possible to assess the manifestation and dynamics of individual behavioural elements and the level of emotional–behavioural activity in rats.

The «Open Field» setup is a circular chamber (diameter 97 cm) with opaque walls of 42 cm height. The floor is divided into sectors for easy visual registration. In addition, there are holes in the floor with a diameter of 2 cm, imitating rat holes.

The rats were divided into 3 groups, 8 animals each: 1—rats, which had intranasal instillation with haloperidol at a dose of 0.5 mg/kg; 2—rats, which had instillation of haloperidol (0.5 mg/kg) in combination with nanoparticles; the control group of rats, which received intranasal instillation of saline. Intranasal administration was carried out 30 min before the start of testing using the technique similar to the one used in the catalepsy test.

Each rat was placed in the centre of the open field and for 3 min the following was recorded: (a) locomotor activity (the number of crossed lines), which reflects the nonspecific level of excitement or depression; (b) exploratory activity (the number of holes examined).

The fixation of behavioural changes was carried out using a digital video system with the Ethovision XT program with an automatic method for analysing tracks on a Noldus device (Netherlands). This made it possible to have a more accurate quantitative assessment of behavioural differences in rats. GraphPad Prism 8.0.1 software was used with the Student’s *t*-test for the analysis and statistical processing of the behavioural data.

## 3. Results and Discussions

### 3.1. Haloperidol Loading

**Figure 1:**
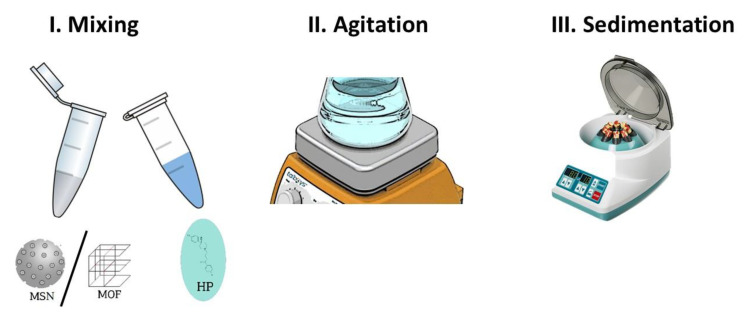
Preparation protocol for haloperidol loading to MSN/MOF nanoparticles.

The MOFs and MSNs were loaded with haloperidol using the following protocol: MOF/MSM methanol solutions in the range of 1–4 mg/mL were mixed with 1 mg/mL haloperidol solution in methanol (Figure 1). The mixture was left for 24 h on a mixing stand. The solution was centrifuged at 13,800 rpm as a next step and supernatant was taken for UV analysis. The concentration of haloperidol was determined from UV–vis calibration curve plotted for the absorbance band of pure haloperidol in methanol at 246 nm (Figure S1 in supplementary material). This preparation protocol was optimised for haloperidol loading; other solvents such as acetone, DMSO, ethanol and different mixing times were tried. The preparation of Eudragit/Brij98 nanoparticles with loaded haloperidol was described above.

**Figure 2:**
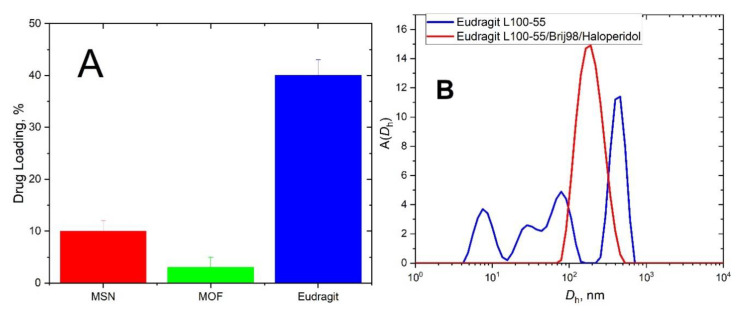
(**A**) Drug loading into MOF, MSN, and hybrid Eudragit/Brij98 nanoparticles. (**B**) The intensity-weighted distribution functions over hydrodynamic diameter *D*_h_ for unloaded at pH = 11 and haloperidol-loaded Eudragit 100-55/Brij98 nanoparticles at pH = 3.

Eudragit L100-55 copolymer and polymeric surfactant Brij98 nanoparticles show the best results in terms of loading efficiency and stability of haloperidol-loaded nanoparticles (Figure 2A). Metal–organic framework ZIF8 failed to encapsulate haloperidol whereas mesoporous silica held the second place showing a bit higher encapsulation ability.

The success of haloperidol loading into Eudragit nanoparticles co-incided with a dramatic change of *D*_h_ distribution function measured by DLS (Figure 2B). In basic conditions, the DLS gave a broad, multi-modal distribution. This converts to a single-mode distribution after addition of haloperidol and adjustment of pH to acidic environment (Figure 2B). This result provides a direct evidence of haloperidol encapsulation inside Eudragit 100-55/Brij98 nanoparticles. The presence of unloaded haloperidol would be immediately manifested on a distribution function by the appearance of additional peak due to drug aggregates/precipitation. The haloperidol-loaded Eudragit100-55/Brij98 nanoparticles showed enhanced stability over several weeks. No sign of precipitation was observed neither by eye nor DLS inspection.

Having established the low haloperidol (HP) loading into MSNs and MOF particles, a new question has arisen—what could be the reason for weak drug loading? There are two most likely explanations for this phenomenon—(1) the incompatibility of HP with the pore sizes of MOF and MSN and (2) weak surface binding. Indeed, the pore volume on the surface of MSN controls drug loading. The pore volume varies from 0.9 to 2.0 cm^3^/g [18]. High drug amounts absorbed into the MSN matrix usually correlate with a larger pore volume. We can assume as a working hypothesis that HP is incompatible with MSN/MOF pores. Applied very simple and generally, this hypothesis means that HP molecules are bigger than MSN/MOF pores. However, there is a growing mass of evidence in drug delivery area that doxorubicin drug with higher molecular dimensions in compassion with HP can be successfully loaded in MSNs and MOFs nanoparticles [15,16,49,50,51,52,53,54].

**Figure 3:**
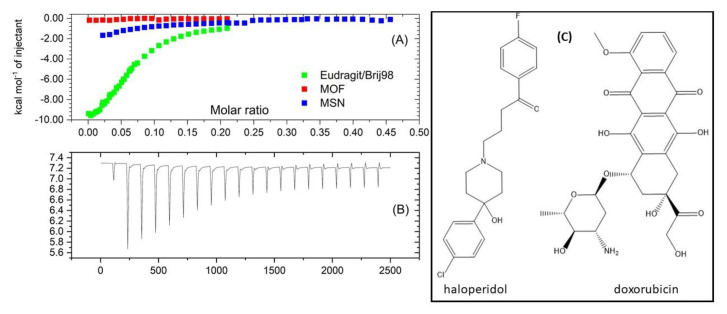
Left: ITC enthalpograms for the titration of Haloperidol in MSN, MOF, and Eudragit/Brij98 solutions (**A**) together with a typical titration curve of haloperidol injection to the solution of Eudragit/Brij98 (**B**). Right: the chemical structures of haloperidol and doxorubicin (**C**).

Thus, our primarily hypothesis should be discarded, and we have to pay attention to the binding properties of haloperidol. Speaking in terms of hydrogen bond formation capability, doxorubicin has superior properties over haloperidol having 6 hydrogen bond donor and 12 hydrogen bond acceptor centres in comparison with 1 and 4 donor and acceptor centres for haloperidol, respectively (Figure 3, right). Such a striking discrepancy indirectly justifies possible low HP binding to the MSN/MOF surface. We performed a series of ITC experiments to clarify the difference between the interactions of haloperidol and different hybrid nanoparticles tested in the present manuscript (Figure 3, left). With all parameters are equal, titration of haloperidol to Eudragit solution shows much higher calorimetric effect in comparison with the titration to MOFs or MSNs (Figure 3A). In line with drug loading results, MOFs show the weakest binding, if any. MSNs possess intermediate binding with haloperidol (Figure 3A). As we focused in this report on the comparative study of the hybrid nanoparticles, the complete thermodynamic analysis of HP- hybrid nanoparticles were out of the scope of this research.

Thus, ITC experiments provide a direct proof that the reason of negligible HP loading to MOFs/MSNs particles is low surface binding possible coming from a lack of hydrogen bonding centres for a HP molecule.

**Figure 4:**
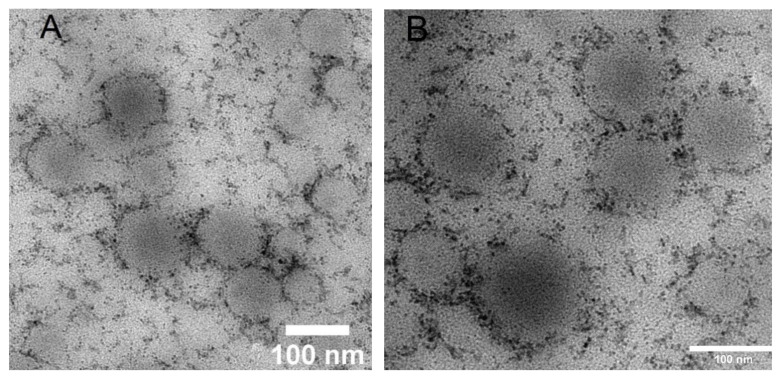
(**A**,**B**) Hybrid nanoparticles Brij98/Eudragit L100-55/haloperidol after preparation.

Given that MOFs and MSNs particles do not encapsulate HP, we focused on the study of Eudragit/Brij98 loaded with HP. TEM-experiments were conducted. It was found by transmission electron microscopy that the hybrid nanoparticles have a spherical shape with core–shell morphology and moderate polydispersity (Figure 4A,B). Taking into consideration the difference in the transmission contrast we attributed the core to hydrophobic domains of Brij98 and Eudragit L100-55 and the shell to partially charged polymeric chains. We could also speculate that HP could be incorporated inside the shell. (Figure 4B). However, we cannot unambiguously identify the location of haloperidol inside of the nanoparticles.

**Figure 5:**
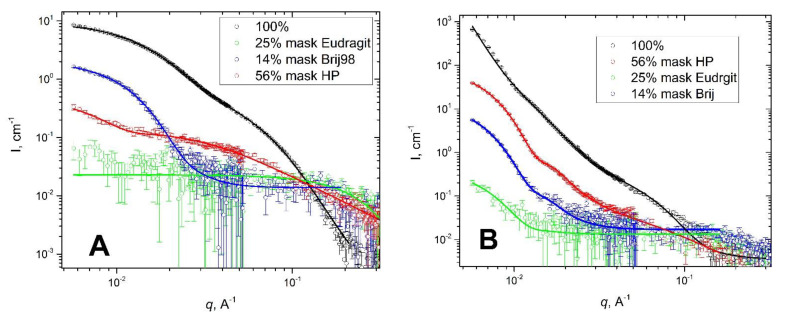
SANS contrast variation data for (**A**) pH = 5; and (**B**) pH = 3; Solid lines are fits.

For this reason, the structure of Eudragit L100-55/Brij98 nanoparticles has been additionally studied by SANS. By contrast variation study we were able to mask either Eudragit L100-55 or Brij98 components (Figure S2). Experiments were performed in different H_2_O-D_2_O mixtures. As one can see from Figure 5, we can specifically suppress the scattering from different parts of a hybrid nanoparticle. To fit the scattering curves, we have used a combination of several basic models: the model of a polydisperse sphere, and the model of a generalized Gaussian coil. The generalized Gaussian coil model [55] (parameters: *R*_g_ —radius of gyration of a polymer chain; *v*—Flory exponent) was taken to describe the decay at middle q range (Supporting Information). The fitting results obtained for both pH 3 and pH 5 systems, Table S1, additionally confirm the core–shell architecture of hybrid nanoparticles composed of Eudragit macromolecules with Brji98 micelles attached to Eudragit chain.

**Figure 6:**
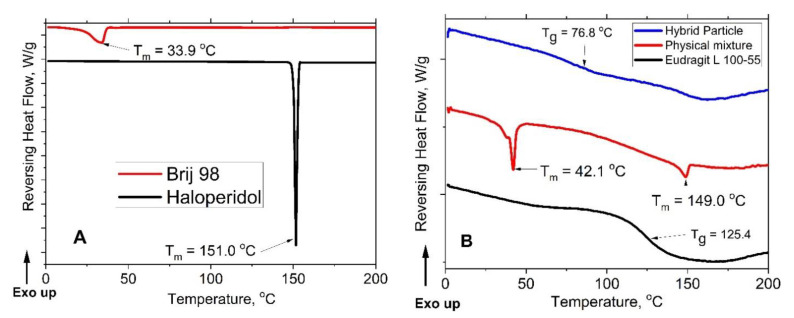
DSC thermograms: (**A**) Brij 98 and haloperidol, and (**B**) L100-55, a physical mixture of haloperidol, Brij 98 and L100-55 and hybrid nanoparticles.

It has been established that loaded drug efficacy, pharmacokinetics and pharmacodynamics are significantly influenced by the state of the drug inside nanoparticles. Using thermal analysis, we examine the question if the haloperidol is crystalline or amorphous. Particles loaded with haloperidol were lyophilized and studied by thermal analysis. The results are presented in Figure 6A,B. The key question that we addressed in this study if we can change the haloperidol state by changing the preparation protocol.

On the mDSC thermogram of dried nanoparticles with haloperidol, the melting peak recorded for the initial crystalline drug form at 151.0 °C (Figure 6A) is absent. However, the pronounced glass transition process at *T*_g_ = 76.8 °C (Figure 6B) indicates that haloperidol is in an amorphous state inside the nanoparticles. *T*_m_ value for the polymer surfactant Brij 98 is 33.9 °C. At the same time, on the thermogram of the physical mixture (Haloperidol, L100-55 and Brij 98), the recorded *T*_m_ value of Brij98 is slightly higher (42.1 °C), while for haloperidol it practically coincides with the *T*_m_ of the individual drug (149.0 °C). mDSC experiments give evidence to the different states of haloperidol in different environment.

**Figure 7:**
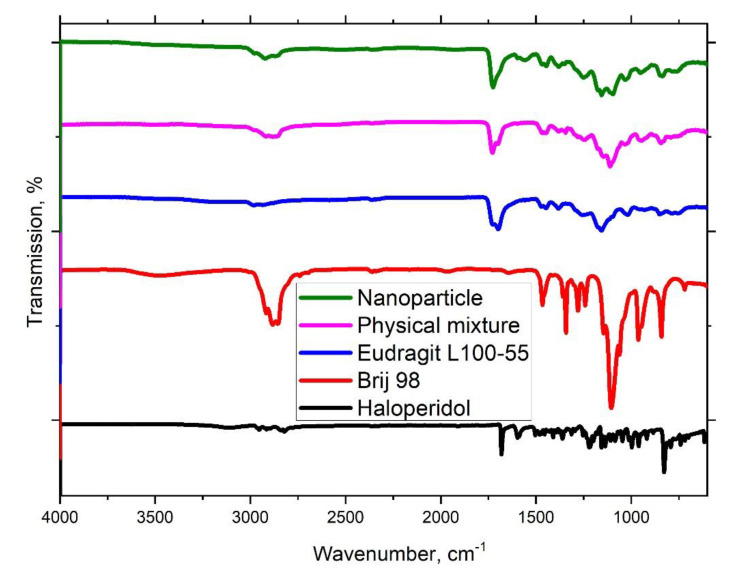
IR spectra of copolymer L100-55, Brij 98, haloperidol, their physical mixture and hybrid nanoparticles.

To elucidate the reasons for the phase transition of a crystalline form into an amorphous one, we studied the IR spectra of hybrid nanoparticles in comparison with a physical mixture similar in composition and individual components (Figure 7).

The IR spectrum of haloperidol has a characteristic band at 1595 cm^−1^ assigned to the stretching vibrations of the bonds in the benzene ring [56]. The presence of this band is also seen in the IR spectra of the physical mixture and haloperidol/L100-55 hybrid nanoparticles, which proves inclusion of the drug. IR spectra of Eudragit^®^ L100-55, physical mixture and haloperidol/L100-55 hybrid nanoparticle are also characterized by the presence of the esterified carboxyl groups band at 1725 cm^−1^ in Eudragit^®^ L100-55 structure. A new characteristic band appears at 1557 cm^−1^ in the IR spectra of haloperidol/L100-55 hybrid nanoparticles, due to the stretching vibration of the carboxylate groups of Eudragit^®^ L100-55 forming the ionic bonds with the protonated nitrogen atom of haloperidol [57]. The chemical interaction of haloperidol with the polyanionic Eudragit^®^ L100-55, most likely, leads to the formation of haloperidol/L100-55 hybrid nanoparticles, in which the drug, due to the strong interactions with the copolymer exists in amorphous form.

Having established the structure of Eudragit/Brij98 nanoparticles loaded with HP, we have focused on therapeutic efficacy of haloperidol. The key question to be addressed here is if HP keeps its antipsychotic effect with solubilization inside the hybrid nanoparticles.

### 3.2. In Vivo Catalepsy Experiments

**Figure 8:**
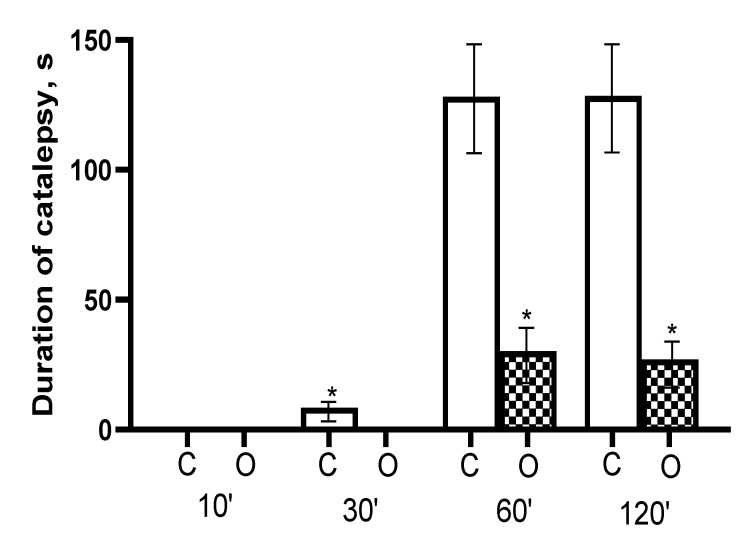
Indicators of the cataleptogenic action of haloperidol after intranasal administration to rats. On the abscissa axis—observation intervals (min) and groups of animals: C—control (haloperidol, 1 mg/kg; n = 8), O—experiment (nanoparticles with haloperidol, 1 mg/kg; n = 8). The ordinate is the duration of catalepsy in seconds. *—statistically significant differences at *p* < 0.05 (t—Student’s test).

**Figure 9:**
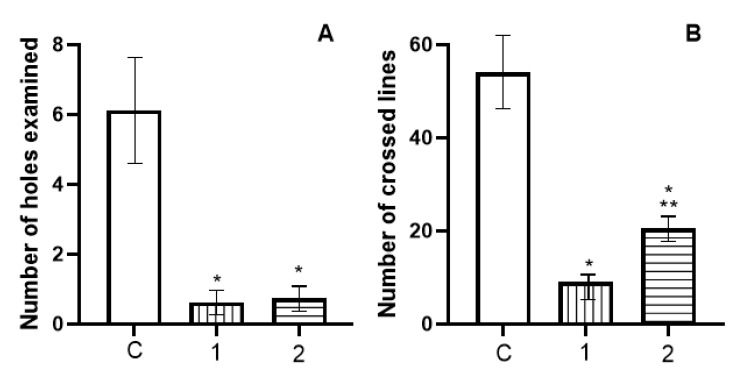
Indicators of research (**A**) and motor (**B**) activity of rats in the «Open field» after intranasal administration of haloperidol. On the abscissa—groups of animals: C—control (saline, intranasally, n = 8); 1—intranasal haloperidol 0.5 mg/kg, n = 8; 2—intranasal haloperidol with nanoparticles, 0.5 mg/kg, n = 8. The ordinate is the number of peeks into the holes (**A**) and the number of line crossings (**B**). *—statistically significant differences in relation to the control group (C) at *p* < 0.05; **—statistically significant differences in relation to group 1 (haloperidol) at *p* < 0.05.

The results of the study of cataleptogenic effect of haloperidol and its formulations with nanoparticles after intranasal administration are shown in Figure 8. Rats did not exhibit any signs of catalepsy 10 min after nasal administration. After 30 min only the commercial haloperidol formulation (used as a control) had some minor catalepsy effects. In 60 min following nasal administration, commercial haloperidol formulation resulted in substantially increased intensity of catalepsy, the time spent by rats on the rod was 128 ± 27 s, whereas the rats instilled with the formulation of haloperidol from nanoparticles exhibited significantly shorter time of 30 ± 11 s (*p* < 0.05). Similar differences between control and nanoparticle formulation was observed 120 min following nasal administration. The study of the behavioural characteristics from haloperidol and its formulations with the intranasal administration using the open field method demonstrated a very similar trend (Figure 9). Intranasal administration of haloperidol to rats caused inhibition of exploratory activity, reducing the number of holes examined, as well as inhibition of motor activity, reducing the number of lines crossed. Rats, which received instillation of haloperidol with nanoparticles displayed higher rates of exploratory and locomotor activity compared to free haloperidol.

Overall, the results of in vivo experiments indicate that intranasal administration of haloperidol in combination with nanoparticles results in more modest effects compared to free drug formulation. This could be related to a number of factors such as predominant deposition of nanoparticles in regions of the nasal cavity that does not favour rapid drug absorption or slower release of drug molecules from the nanoparticles.

One of the possible release mechanisms could be the following: Eudragit L100-55 starts to dissolve in aqueous media at pH > 5.5. The pH value of nanoparticle solution is 3.0–5.0, allowing them to remain stable in aqueous solution. When instilled into the nasal cavity, nanoparticles can be absorbed in the mucus due to the mucoadhesive properties of Eudragit L100-55 [58]. The pH of the nasal mucosa is 5.5–6.5, and it increases in rhinitis to 7.2–8.3 [59]. We assume that conformational changes in the polymer molecules occur in the nasal mucosa caused by an increase in the pH of the medium, which leads to the nanoparticles “unfolding” and the release of haloperidol.

## 4. Conclusions

Hybrid polymer nanoparticles based on Eudragit^®^ L100-55 polyanion and Brij 98 polymer surfactant were made. The resulting nanoparticles self-assemble at a pH below 5.5. Their size can vary depending on the final pH and the ratio of polymer and surfactant. In view of the significant hydrophobicity of haloperidol and, as a consequence, the low solubility of haloperidol in water, it was proposed to use water–organic mixtures (water–acetone, water–methanol, and water–DMSO) to improve the degree of drug loading into nanoparticles. The best results were observed for a mixed solvent of water-acetone and evaporation of acetone using a vacuum rotary evaporator. We have optimized the haloperidol loading into the hybrid Eudragit L100-55/Brij98 nanoparticles as the next step reaching up to 40% loading efficiency with high stability over several months. The internal structure of the hybrid nanoparticles was probed using transmission electron microscopy and contrast variation SANS. The internal structure of hybrid nanoparticles was found to be a the core–shell structure with the inclusion of Brij 98 micelles inside of the hybrid nanoparticle and haloperidol incorporated inside of a hydrophobic core. mDSC studies indicate the encapsulated haloperidol is an in amorphous state inside the Eudragit L100-55/Brij98 nanoparticles. Using a catalepsy and open field animal tests, we demonstrated the prolongation of haloperidol release, which results in a later onset of drug action. The possible molecular mechanisms of haloperidol action and administration routes are discussed.

## Data Availability

Data are contained within this article.

## Supplementary Materials

The following are available online at https://www.mdpi.com/article/10.3390/polym13234189/s1, Figure S1: The calibration plot for the absorbance band of haloperidol in methanol at 246 nm; Figure S2: Contrast matching in the SANS experiments; Table S1: Fitting parameters for Eudragit/Brij98 systems at different pH values.
