# Supplementary material for: Hybrid Nanoparticles for Haloperidol Encapsulation: Quid Est Optimum?
Source: Polymers (Basel). 2021 Nov 30;13(23):4189. doi: 10.3390/polym13234189 (PMC8659838; doi:10.3390/polym13234189)
Supplement: Supplementary file 1 [file polymers-13-04189-s001.zip › polymers-1455316-supplementary.pdf]

Hybrid Nanoparticles for Haloperidol encapsulation: *Quid est optimum*?

Sergey K. Filippov, Ramil R. Khusnutdinov, Wali Inham, Chang Liu, Christopher Garvey, Dmitry O. Nikitin, Irina I. Semina, Shamil F. Nasibullin, Vitaliy V. Khutoryanskiy, Hongbo Zhang, Rouslan I. Moustafine

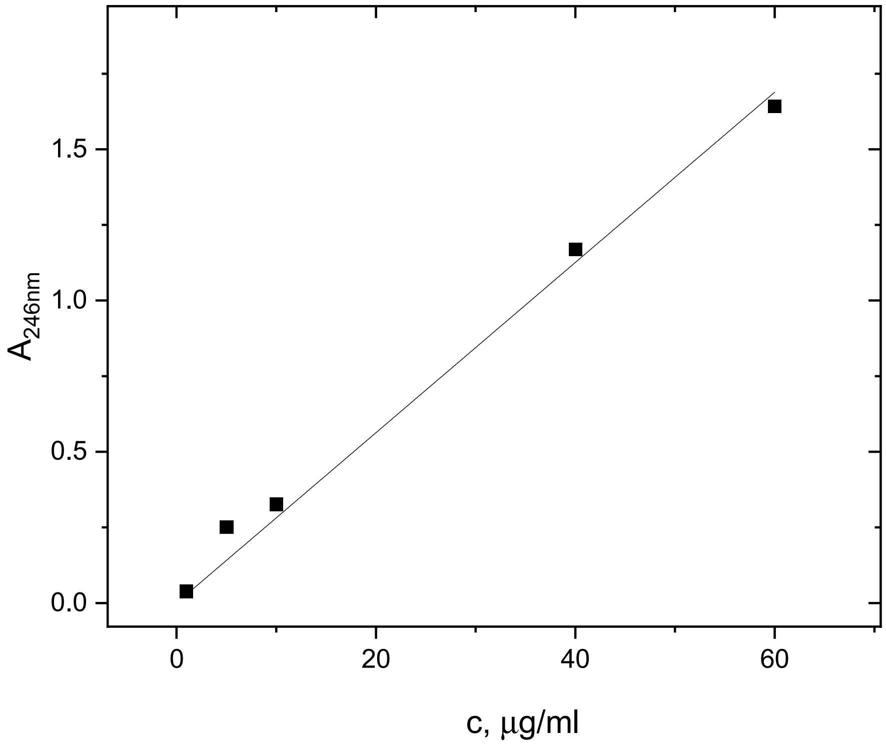

Figure S1. The calibration plot for the absorbance band of haloperidol in methanol at 246 nm.

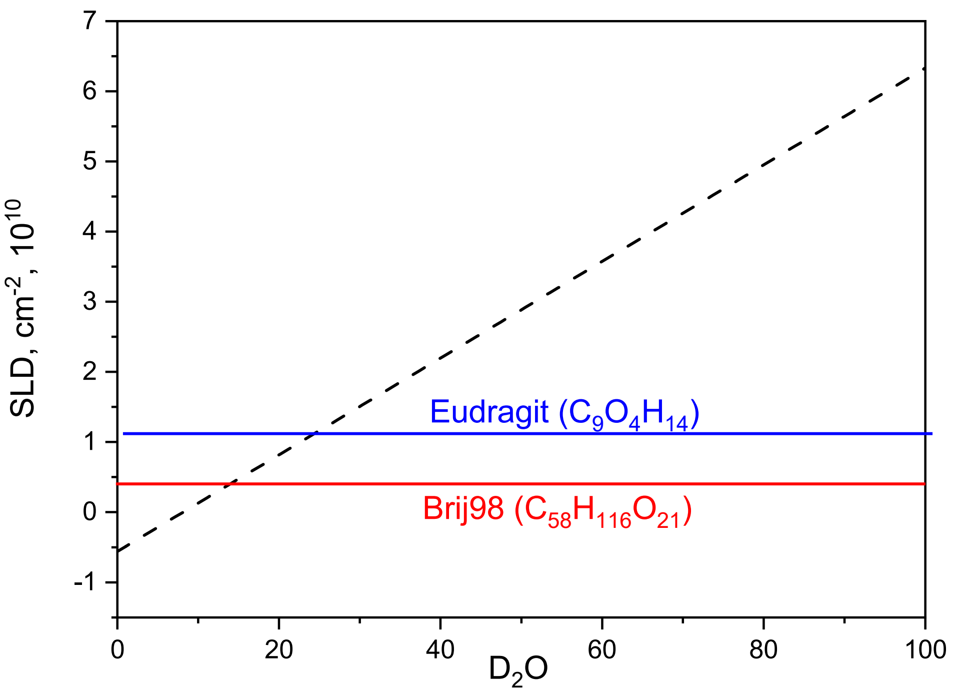

Figure S2. Contrast matching in the SANS experiments.

The generalized Gaussian coil model.

The model of generalized Gaussian coil was used for fitting at basic pH values. The scattering function for the generalized Gaussian coil could be described by Eq. 1 as

$$I(q) = I_0 \left[\frac{U^{\frac{1}{2\nu}} \Gamma(\frac{1}{2\nu}) - \Gamma(\frac{1}{\nu}) - U^{\frac{1}{2\nu}} \Gamma(\frac{1}{2\nu}, U) + \Gamma(\frac{1}{\nu}, U)}{\nu U^{\frac{1}{\nu}}} \right] \quad (1)$$

where U = (2ν + 1)(2ν + 2)$q^2 R^2_g/6$; Γ(a,x) is unnormalized incomplete Gamma Function and Γ(a) is the Gamma function; ν is the excluded volume parameter from the Flory mean field theory.

Table S1. Fitting parameters for Eudragit/Brij98 systems at different pH values

pH	$\%D_2O$	big spherical particles		small spherical particles		generalized Gaussian coil	
		R_{big}, Å	sigma	$R_{s\ mall}$, Å		R_g, Å	ν
3	14	322±1	0.21±0.01				
3	25	300±1	0.21±0.02				
3	56	304±1	0.20±0.02			25.9±0.1	0.5±0.2
3	100	95.8±0.2	0.53±0.03	27.3±0.02	0.3		
5	14	142±1	0.28±0.04				
5	25			9.3±0.01	0.1±0.03		
5	56					40.6±0.1	0.65±0.01
5	100	52.2±0.2	0.58±0.04	17.5±0.02	0.46±0.03		
